# {2-Morpholino-*N*-[1-(2-pyrid­yl)ethyl­idene]ethanamine-κ^3^ *N*,*N*′,*N*′′}bis­(thio­cyanato-κ*N*)copper(II)

**DOI:** 10.1107/S1600536810050889

**Published:** 2010-12-11

**Authors:** Nura Suleiman Gwaram, Nurul Azimah Ikmal Hisham, Hamid Khaledi, Hapipah Mohd Ali

**Affiliations:** a Department of Chemistry, University of Malaya, 50603 Kuala Lumpur, Malaysia

## Abstract

In the title compound, [Cu(NCS)_2_(C_13_H_19_N_3_O)], the Cu^II^ ion is five-coordinated by the *N*,*N*′,*N*′′-tridentate Schiff base and the N atoms of two isothio­cyanate ligands in a square-pyramidal geometry. In the crystal, C—H⋯N, C—H⋯O and C—H⋯S inter­actions link adjacent mol­ecules into layers parallel to the *ac* plane. A weak inter­molecular π–π inter­action occurs between the aromatic rings with a centroid–centroid distance of 3.9412 (9) Å.

## Related literature

For related structures of Cu(II) complexes, see: Drew *et al.* (2009 ▶); You *et al.* (2006 ▶); Yue *et al.* (2005 ▶).
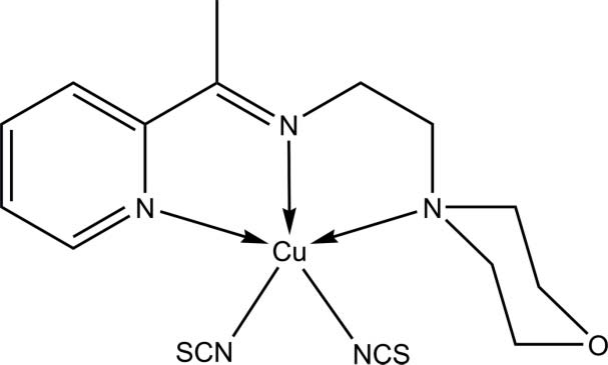


## Experimental

### 

#### Crystal data

[Cu(NCS)_2_(C_13_H_19_N_3_O)]
*M* *_r_* = 413.01
Monoclinic, 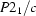

*a* = 10.6912 (1) Å
*b* = 14.0350 (2) Å
*c* = 12.2530 (2) Å
β = 92.203 (1)°
*V* = 1837.22 (4) Å^3^
*Z* = 4
Mo *K*α radiation
μ = 1.43 mm^−1^
*T* = 100 K
0.41 × 0.39 × 0.08 mm

#### Data collection

Bruker APEXII CCD diffractometer
Absorption correction: multi-scan (*SADABS*; Sheldrick, 1996 ▶) *T*_min_ = 0.592, *T*_max_ = 0.894
16491 measured reflections
4007 independent reflections
3593 reflections with *I* > 2σ(*I*)
*R*_int_ = 0.024

#### Refinement

*R*[*F* ^2^ > 2σ(*F* ^2^)] = 0.023
*wR*(*F* ^2^) = 0.061
*S* = 1.08
4007 reflections
218 parameters
2 restraints
H-atom parameters constrained
Δρ_max_ = 0.37 e Å^−3^
Δρ_min_ = −0.26 e Å^−3^

### 

Data collection: *APEX2* (Bruker, 2007 ▶); cell refinement: *SAINT* (Bruker, 2007 ▶); data reduction: *SAINT*; program(s) used to solve structure: *SHELXS97* (Sheldrick, 2008 ▶); program(s) used to refine structure: *SHELXL97* (Sheldrick, 2008 ▶); molecular graphics: *X-SEED* (Barbour, 2001 ▶); software used to prepare material for publication: *SHELXL97* and *publCIF* (Westrip, 2010 ▶).

## Supplementary Material

Crystal structure: contains datablocks I, global. DOI: 10.1107/S1600536810050889/is2639sup1.cif

Structure factors: contains datablocks I. DOI: 10.1107/S1600536810050889/is2639Isup2.hkl

Additional supplementary materials:  crystallographic information; 3D view; checkCIF report

## Figures and Tables

**Table 1:** Hydrogen-bond geometry (Å, °)

*D*—H⋯*A*	*D*—H	H⋯*A*	*D*⋯*A*	*D*—H⋯*A*
C4—H4⋯O1^i^	0.95	2.40	3.211 (2)	144
C7—H7*B*⋯O1^i^	0.98	2.33	3.248 (2)	155
C7—H7*C*⋯S2^ii^	0.98	2.83	3.7021 (18)	149
C8—H8*B*⋯N5^ii^	0.99	2.55	3.367 (2)	140
C13—H13*A*⋯N4	0.99	2.58	3.181 (2)	119

Symmetry codes: (i) 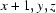; (ii) 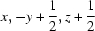.

**Fig. 1.**
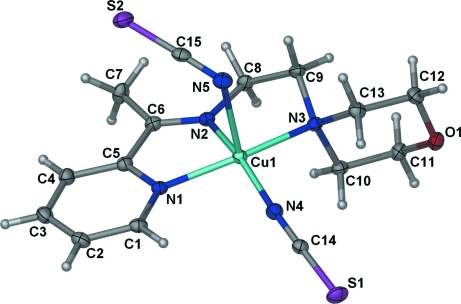
Thermal ellipsoid plot of the title compound at the 50% probability level. Hydrogen atoms are drawn as spheres of arbitrary radii.

**Fig. 2.**
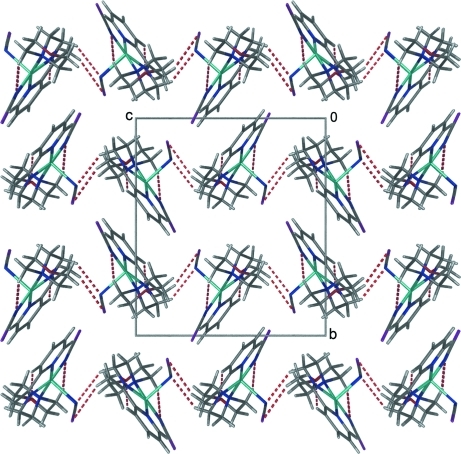
The packing diagram of the title compound, viewed down the crystallographic a axis.

## supplementary crystallographic information

### Comment

The title compound is a mixed-ligand copper(II) complex with isothiocyanate and the Schiff base 2-morpholino-*N*-[1-(2-pyridyl)ethylidene]ethanamine. The geometry of the complex is slightly distorted square-pyramidal (τ = 0.05) with the *N,N',N"*-tridentate Schiff base and one SCN ligand at the basal positions, while the apical position is occupied by a second SCN ligand. This arrangement has been observed in some other mixed-ligand copper(II) complexes (Drew *et al.*, 2009; You *et al.*, 2006; Yue *et al.*, 2005). In the crystal structure, the C—H···N, C—H···O and C—H···S interactions within the range for normal hydrogen bonds link the adjacent molecules into layers parallel to the *ac* plane (Fig. 2). An intramolecular C—H···N hydrogen bonding is also observed. Moreover, the aromatic rings of each two molecules related by the symmetry -*x* + 2, -*y*, -*z* + 1, are arranged in an antiparallel manner with centroid-centroid separation of 3.9412 (9) Å, indicative of a weak π–π interaction.

### Experimental

A mixture of 2-acetylpyridine (0.20 g, 1.65 mmol) and 4-(2-aminoethyl)morpholine (0.21 g, 1.65 mmol) in ethanol (20 ml) was refluxed for 2 hr followed by addition of a solution of copper(II) acetate monohydrate (0.33 g, 1.65 mmol) and sodium thiocyanete (0.134 g, 1.65 mmol) in a minimum amount of ethanol. The resulting solution was refluxed for 30 min, then left at room temperature. The crystals of the title complex were obtained in a week.

### Refinement

The hydrogen atoms were placed at calculated positions (C—H 0.95–0.99 Å) and were treated as riding on their parent atoms with *U*_iso_(H) set to 1.2 or 1.5*U*_eq_(C). An additional rigid-bond type restraint (*DELU* in *SHELXL97*) was placed on the displacement parameters of S1 and C14; S2 and C15.

### Crystal data

**Table:**

[Cu(NCS)_2_(C_13_H_19_N_3_O)]	*F*(000) = 852
*M**_r_* = 413.01	*D*_x_ = 1.493 Mg m^−^^3^
Monoclinic, *P*2_1_/*c*	Mo *K*α radiation, λ = 0.71073 Å
Hall symbol: -P 2ybc	Cell parameters from 7443 reflections
*a* = 10.6912 (1) Å	θ = 2.2–30.7°
*b* = 14.0350 (2) Å	µ = 1.43 mm^−^^1^
*c* = 12.2530 (2) Å	*T* = 100 K
β = 92.203 (1)°	Plate, blue
*V* = 1837.22 (4) Å^3^	0.41 × 0.39 × 0.08 mm
*Z* = 4	

### Data collection

**Table:**

Bruker APEXII CCD diffractometer	4007 independent reflections
Radiation source: fine-focus sealed tube	3593 reflections with *I* > 2σ(*I*)
graphite	*R*_int_ = 0.024
φ and ω scans	θ_max_ = 27.0°, θ_min_ = 2.2°
Absorption correction: multi-scan (*SADABS*; Sheldrick, 1996)	*h* = −13→13
*T*_min_ = 0.592, *T*_max_ = 0.894	*k* = −17→17
16491 measured reflections	*l* = −15→15

### Refinement

**Table:**

Refinement on *F*^2^	Primary atom site location: structure-invariant direct methods
Least-squares matrix: full	Secondary atom site location: difference Fourier map
*R*[*F*^2^ > 2σ(*F*^2^)] = 0.023	Hydrogen site location: inferred from neighbouring sites
*wR*(*F*^2^) = 0.061	H-atom parameters constrained
*S* = 1.08	*w* = 1/[σ^2^(*F*_o_^2^) + (0.0264*P*)^2^ + 0.8835*P*] where *P* = (*F*_o_^2^ + 2*F*_c_^2^)/3
4007 reflections	(Δ/σ)_max_ = 0.001
218 parameters	Δρ_max_ = 0.37 e Å^−^^3^
2 restraints	Δρ_min_ = −0.26 e Å^−^^3^

### Special details

**Table:**

Geometry. All e.s.d.'s (except the e.s.d. in the dihedral angle between two l.s. planes) are estimated using the full covariance matrix. The cell e.s.d.'s are taken into account individually in the estimation of e.s.d.'s in distances, angles and torsion angles; correlations between e.s.d.'s in cell parameters are only used when they are defined by crystal symmetry. An approximate (isotropic) treatment of cell e.s.d.'s is used for estimating e.s.d.'s involving l.s. planes.
Refinement. Refinement of *F*^2^ against ALL reflections. The weighted *R*-factor *wR* and goodness of fit *S* are based on *F*^2^, conventional *R*-factors *R* are based on *F*, with *F* set to zero for negative *F*^2^. The threshold expression of *F*^2^ > σ(*F*^2^) is used only for calculating *R*-factors(gt) *etc*. and is not relevant to the choice of reflections for refinement. *R*-factors based on *F*^2^ are statistically about twice as large as those based on *F*, and *R*- factors based on ALL data will be even larger.

### Fractional atomic coordinates and isotropic or equivalent isotropic displacement parameters (Å^2^)

**Table:**

	*x*	*y*	*z*	*U*_iso_*/*U*_eq_	
Cu1	0.828576 (17)	0.205404 (13)	0.447661 (15)	0.01426 (6)	
S1	0.54717 (4)	−0.02085 (3)	0.29655 (4)	0.02655 (11)	
S2	1.11219 (4)	0.39163 (3)	0.33440 (4)	0.02849 (11)	
O1	0.42152 (11)	0.29240 (8)	0.55167 (10)	0.0219 (3)	
N1	0.99124 (12)	0.13295 (9)	0.43945 (10)	0.0152 (3)	
N2	0.92988 (12)	0.27488 (9)	0.55693 (10)	0.0153 (3)	
N3	0.68518 (12)	0.28625 (9)	0.50715 (11)	0.0155 (3)	
N4	0.72317 (13)	0.11286 (10)	0.37153 (11)	0.0194 (3)	
N5	0.87234 (14)	0.31121 (10)	0.31479 (11)	0.0212 (3)	
C1	1.01523 (15)	0.05728 (11)	0.37750 (12)	0.0176 (3)	
H1	0.9486	0.0289	0.3355	0.021*	
C2	1.13462 (16)	0.01887 (12)	0.37269 (13)	0.0204 (3)	
H2	1.1497	−0.0345	0.3273	0.024*	
C3	1.23136 (16)	0.05957 (12)	0.43502 (14)	0.0216 (3)	
H3	1.3138	0.0346	0.4328	0.026*	
C4	1.20628 (15)	0.13735 (12)	0.50084 (13)	0.0200 (3)	
H4	1.2711	0.1659	0.5449	0.024*	
C5	1.08541 (15)	0.17262 (11)	0.50118 (12)	0.0163 (3)	
C6	1.04657 (15)	0.25572 (11)	0.56749 (12)	0.0166 (3)	
C7	1.13919 (16)	0.30782 (13)	0.63899 (14)	0.0233 (4)	
H7A	1.1033	0.3688	0.6612	0.035*	
H7B	1.2152	0.3196	0.5989	0.035*	
H7C	1.1600	0.2694	0.7040	0.035*	
C8	0.86732 (15)	0.35203 (11)	0.61333 (13)	0.0182 (3)	
H8A	0.9224	0.4087	0.6188	0.022*	
H8B	0.8471	0.3318	0.6880	0.022*	
C9	0.74783 (15)	0.37566 (11)	0.54728 (13)	0.0184 (3)	
H9A	0.6903	0.4117	0.5934	0.022*	
H9B	0.7681	0.4163	0.4843	0.022*	
C10	0.62870 (15)	0.23026 (11)	0.59640 (13)	0.0178 (3)	
H10A	0.6913	0.2230	0.6575	0.021*	
H10B	0.6070	0.1658	0.5688	0.021*	
C11	0.51185 (15)	0.27710 (12)	0.63883 (13)	0.0204 (3)	
H11A	0.4754	0.2359	0.6949	0.024*	
H11B	0.5344	0.3388	0.6734	0.024*	
C12	0.47049 (16)	0.35294 (12)	0.47023 (14)	0.0223 (3)	
H12A	0.4932	0.4154	0.5029	0.027*	
H12B	0.4059	0.3637	0.4115	0.027*	
C13	0.58532 (15)	0.30782 (12)	0.42254 (13)	0.0191 (3)	
H13A	0.5604	0.2481	0.3847	0.023*	
H13B	0.6193	0.3515	0.3676	0.023*	
C14	0.65038 (15)	0.05637 (11)	0.34076 (12)	0.0165 (3)	
C15	0.97181 (16)	0.34563 (11)	0.32179 (13)	0.0190 (3)	

### Atomic displacement parameters (Å^2^)

**Table:**

	*U*^11^	*U*^22^	*U*^33^	*U*^12^	*U*^13^	*U*^23^
Cu1	0.01509 (10)	0.01341 (10)	0.01417 (10)	−0.00117 (7)	−0.00099 (7)	−0.00142 (7)
S1	0.0282 (2)	0.0203 (2)	0.0307 (2)	−0.00934 (17)	−0.00451 (18)	−0.00294 (18)
S2	0.0273 (2)	0.0211 (2)	0.0369 (3)	−0.00521 (17)	−0.00168 (19)	0.00639 (19)
O1	0.0166 (6)	0.0241 (6)	0.0251 (6)	−0.0018 (5)	0.0010 (5)	0.0008 (5)
N1	0.0182 (7)	0.0140 (6)	0.0135 (6)	−0.0012 (5)	0.0005 (5)	0.0014 (5)
N2	0.0179 (7)	0.0143 (6)	0.0137 (6)	−0.0018 (5)	0.0007 (5)	−0.0003 (5)
N3	0.0163 (7)	0.0152 (6)	0.0150 (6)	−0.0016 (5)	−0.0008 (5)	0.0012 (5)
N4	0.0196 (7)	0.0183 (7)	0.0200 (7)	−0.0007 (5)	−0.0014 (5)	−0.0012 (6)
N5	0.0269 (8)	0.0199 (7)	0.0167 (7)	0.0000 (6)	0.0007 (6)	0.0027 (5)
C1	0.0235 (8)	0.0150 (7)	0.0141 (7)	−0.0014 (6)	−0.0007 (6)	0.0014 (6)
C2	0.0271 (9)	0.0164 (8)	0.0178 (8)	0.0031 (6)	0.0014 (6)	−0.0001 (6)
C3	0.0210 (8)	0.0202 (8)	0.0236 (8)	0.0032 (6)	0.0017 (7)	0.0033 (7)
C4	0.0182 (8)	0.0205 (8)	0.0212 (8)	−0.0008 (6)	−0.0016 (6)	0.0011 (7)
C5	0.0186 (8)	0.0162 (7)	0.0142 (7)	−0.0019 (6)	0.0007 (6)	0.0013 (6)
C6	0.0197 (8)	0.0168 (7)	0.0134 (7)	−0.0025 (6)	0.0013 (6)	−0.0003 (6)
C7	0.0183 (8)	0.0284 (9)	0.0231 (8)	−0.0039 (7)	−0.0007 (7)	−0.0088 (7)
C8	0.0192 (8)	0.0170 (7)	0.0184 (8)	−0.0017 (6)	0.0010 (6)	−0.0053 (6)
C9	0.0191 (8)	0.0140 (7)	0.0223 (8)	−0.0018 (6)	0.0023 (6)	−0.0015 (6)
C10	0.0194 (8)	0.0162 (7)	0.0178 (8)	−0.0020 (6)	−0.0001 (6)	0.0018 (6)
C11	0.0187 (8)	0.0229 (8)	0.0196 (8)	−0.0028 (6)	0.0013 (6)	−0.0002 (7)
C12	0.0212 (9)	0.0201 (8)	0.0255 (9)	0.0018 (6)	−0.0015 (7)	0.0036 (7)
C13	0.0199 (8)	0.0207 (8)	0.0164 (8)	0.0011 (6)	−0.0026 (6)	0.0019 (6)
C14	0.0191 (8)	0.0150 (7)	0.0154 (7)	0.0008 (5)	−0.0001 (6)	0.0005 (6)
C15	0.0276 (8)	0.0142 (7)	0.0154 (7)	0.0016 (6)	0.0013 (6)	0.0029 (6)

### Geometric parameters (Å, °)

**Table:**

Cu1—N4	1.9356 (14)	C3—H3	0.9500
Cu1—N2	1.9506 (13)	C4—C5	1.384 (2)
Cu1—N1	2.0204 (13)	C4—H4	0.9500
Cu1—N3	2.0627 (13)	C5—C6	1.490 (2)
Cu1—N5	2.2657 (14)	C6—C7	1.489 (2)
S1—C14	1.6247 (16)	C7—H7A	0.9800
S2—C15	1.6357 (18)	C7—H7B	0.9800
O1—C12	1.425 (2)	C7—H7C	0.9800
O1—C11	1.428 (2)	C8—C9	1.522 (2)
N1—C1	1.336 (2)	C8—H8A	0.9900
N1—C5	1.355 (2)	C8—H8B	0.9900
N2—C6	1.278 (2)	C9—H9A	0.9900
N2—C8	1.460 (2)	C9—H9B	0.9900
N3—C13	1.4904 (19)	C10—C11	1.521 (2)
N3—C10	1.493 (2)	C10—H10A	0.9900
N3—C9	1.4966 (19)	C10—H10B	0.9900
N4—C14	1.164 (2)	C11—H11A	0.9900
N5—C15	1.168 (2)	C11—H11B	0.9900
C1—C2	1.389 (2)	C12—C13	1.518 (2)
C1—H1	0.9500	C12—H12A	0.9900
C2—C3	1.385 (2)	C12—H12B	0.9900
C2—H2	0.9500	C13—H13A	0.9900
C3—C4	1.390 (2)	C13—H13B	0.9900
			
N4—Cu1—N2	164.55 (6)	C6—C7—H7B	109.5
N4—Cu1—N1	97.15 (5)	H7A—C7—H7B	109.5
N2—Cu1—N1	80.18 (5)	C6—C7—H7C	109.5
N4—Cu1—N3	96.46 (5)	H7A—C7—H7C	109.5
N2—Cu1—N3	83.28 (5)	H7B—C7—H7C	109.5
N1—Cu1—N3	161.32 (5)	N2—C8—C9	107.46 (12)
N4—Cu1—N5	103.06 (5)	N2—C8—H8A	110.2
N2—Cu1—N5	92.35 (5)	C9—C8—H8A	110.2
N1—Cu1—N5	95.29 (5)	N2—C8—H8B	110.2
N3—Cu1—N5	94.03 (5)	C9—C8—H8B	110.2
C12—O1—C11	110.92 (12)	H8A—C8—H8B	108.5
C1—N1—C5	119.27 (14)	N3—C9—C8	110.35 (12)
C1—N1—Cu1	127.79 (11)	N3—C9—H9A	109.6
C5—N1—Cu1	112.82 (10)	C8—C9—H9A	109.6
C6—N2—C8	124.83 (13)	N3—C9—H9B	109.6
C6—N2—Cu1	118.59 (11)	C8—C9—H9B	109.6
C8—N2—Cu1	116.31 (10)	H9A—C9—H9B	108.1
C13—N3—C10	108.51 (12)	N3—C10—C11	112.55 (13)
C13—N3—C9	110.96 (12)	N3—C10—H10A	109.1
C10—N3—C9	112.90 (12)	C11—C10—H10A	109.1
C13—N3—Cu1	112.84 (10)	N3—C10—H10B	109.1
C10—N3—Cu1	107.24 (9)	C11—C10—H10B	109.1
C9—N3—Cu1	104.36 (9)	H10A—C10—H10B	107.8
C14—N4—Cu1	169.48 (13)	O1—C11—C10	110.49 (13)
C15—N5—Cu1	115.52 (12)	O1—C11—H11A	109.6
N1—C1—C2	121.97 (15)	C10—C11—H11A	109.6
N1—C1—H1	119.0	O1—C11—H11B	109.6
C2—C1—H1	119.0	C10—C11—H11B	109.6
C3—C2—C1	118.98 (15)	H11A—C11—H11B	108.1
C3—C2—H2	120.5	O1—C12—C13	110.18 (13)
C1—C2—H2	120.5	O1—C12—H12A	109.6
C2—C3—C4	119.16 (15)	C13—C12—H12A	109.6
C2—C3—H3	120.4	O1—C12—H12B	109.6
C4—C3—H3	120.4	C13—C12—H12B	109.6
C5—C4—C3	118.94 (15)	H12A—C12—H12B	108.1
C5—C4—H4	120.5	N3—C13—C12	112.65 (13)
C3—C4—H4	120.5	N3—C13—H13A	109.1
N1—C5—C4	121.67 (15)	C12—C13—H13A	109.1
N1—C5—C6	114.16 (13)	N3—C13—H13B	109.1
C4—C5—C6	124.17 (14)	C12—C13—H13B	109.1
N2—C6—C7	125.52 (15)	H13A—C13—H13B	107.8
N2—C6—C5	113.64 (14)	N4—C14—S1	178.86 (15)
C7—C6—C5	120.83 (14)	N5—C15—S2	178.35 (15)
C6—C7—H7A	109.5		

### Hydrogen-bond geometry (Å, °)

**Table:**

*D*—H···*A*	*D*—H	H···*A*	*D*···*A*	*D*—H···*A*
C4—H4···O1^i^	0.95	2.40	3.211 (2)	144
C7—H7B···O1^i^	0.98	2.33	3.248 (2)	155
C7—H7C···S2^ii^	0.98	2.83	3.7021 (18)	149
C8—H8B···N5^ii^	0.99	2.55	3.367 (2)	140
C13—H13A···N4	0.99	2.58	3.181 (2)	119

Symmetry codes: (i) *x*+1, *y*, *z*; (ii) *x*, −*y*+1/2, *z*+1/2.

## References

[bb1] Barbour, L. J. (2001). *J. Supramol. Chem.* **1**, 189–191.

[bb2] Bruker (2007). *APEX2* and *SAINT* Bruker AXS Inc., Madison, Wisconsin, USA.

[bb3] Drew, M. G. B., Das, D., De, S. & Datta, D. (2009). *Inorg. Chim. Acta*, **362**, 1501–1505.

[bb4] Sheldrick, G. M. (1996). *SADABS* University of Göttingen, Germany.

[bb5] Sheldrick, G. M. (2008). *Acta Cryst.* A**64**, 112–122. 10.1107/S0108767307043930 18156677

[bb6] Westrip, S. P. (2010). *J. Appl. Cryst.* **43**, 920–925.

[bb7] You, Z.-L., Wang, J. & Han, X. (2006). *Acta Cryst.* E**62**, m860–m861.

[bb8] Yue, G.-R., Xu, X.-J., Shi, Y.-Z. & Feng, L. (2005). *Acta Cryst.* E**61**, m693–m694.

